# Antioxidant capacity of *calendula officinalis* flowers extract and prevention of radiation induced oropharyngeal mucositis in patients with head and neck cancers: a randomized controlled clinical study

**DOI:** 10.1186/2008-2231-21-18

**Published:** 2013-03-07

**Authors:** Neda Babaee, Dariush Moslemi, Mohammad Khalilpour, Fatemeh Vejdani, Yasaman Moghadamnia, Ali Bijani, Mahmoud Baradaran, Mohammad Taghi Kazemi, Asieh Khalilpour, Mahdi Pouramir, Ali Akbar Moghadamnia

**Affiliations:** 1 Department of Oral diseases, School of Dentistry, Babol University of Medical Sciences, Babol, Iran; 2 Division of Radiation Oncology, School of Medicine, Babol University of Medical Sciences, Babol, Iran; 3 School of Basic Sciences, Alzahra University, Tehran, Iran; 4 Non-Communicable Pediatric Disease Research Center, Babol University of Medical Sciences, Babol, Iran; 5 Department of Pharmacology, School of Medicine, Babol University of Medical Sciences, Babol, Iran; 6 Department of Biochemistry, School of Medicine, Babol University of Medical Sciences, Babol, Iran

**Keywords:** Radiotherapy-induced oral mucositis, OMAS (oral mucositis assessment scale), *Calendula officinalis*, Antioxidant capacity, Gel formulation

## Abstract

This study was designed to determine the effect of *Calendula officinalis* flowers extract mouthwash as oral gel on radiation-induced oropharyngeal mucositis (OM) in patients with head-and-neck cancer. Forty patients with neck and head cancers under radiotherapy or concurrent chemoradiotherapy protocols were randomly assigned to receive either 2% calendula extract mouthwash or placebo (20 patients in each group). Patients were treated with telecobalt radiotherapy at conventional fractionation (200 cGy/fraction, five fractions weekly, 30–35 fractions within 4–7 weeks). The oropharyngeal mucositis was evaluated by two clinical investigators (a radiation oncologist and a dentist), using the oral mucositis assessment scale (OMAS). Trying to find out the possible mechanism of action of the treatment, total antioxidant, polyphenol and flavonoid contents, and quercetin concentration of the mouth wash were measured. *Calendula* mouthwash significantly decreased the intensity of OM compared to placebo at week 2 (score: 5.5 vs. 6.8, p = 0.019), week 3 (score: 8.25 vs. 10.95, p < 0.0001) and week 6 (score: 11.4 vs. 13.35, p = 0.031). Total antioxidant, polyphenol and flavonoid contents and quercetin concentration of the 2% extract were 2353.4 ± 56.5 μM, 313.40 ± 6.52 mg/g, 76.66 ± 23.24 mg/g, and 19.41 ± 4.34 mg/l, respectively. *Calendula* extract gel could be effective on decreasing the intensity of radiotherapy- induced OM during the treatment and antioxidant capacity may be partly responsible for the effect.

## Introduction

One of the most commonly perturbing adverse effect of radiotherapy (RT) in head and neck cancers is oropharyngeal mucositis (OM) which comprises the main dose limiting side effect [1]. The OM has been reported by patients to be as the worst side effect of cancer radiotherapy [2,3]. Triottie et al. stated that the incidence of radiotherapy induced OM in head and neck cancer patients is more than 80% [3]. The OM, as a result of cancer management, is defined as inflammation of oral mucosa, typically manifesting as atrophy, swelling, erythema and ulceration [4]. Up to this date, no approved standard intervention has been found for the prevention or treatment of radiotherapy-induced OM. Several strategies are being actively investigated, which are based on the mechanistic interruption of one or more pathobiological pathways involved in this condition [5]. A large number of treatments and strategies have been studied for OM such as, growth factors, cytokines, antibiotics, anti-inflammatory agents, cryotherapy, mucosal protectors [6,7] and traditional therapy such as pure honey [8] and herbal medicines [9]. As an example, it has been shown that chamomile mouthwash, is fairly effective on reducing post chemotherapy-induced OM [9].

*Calendula officinalis*, commonly known as Marigold, is used in the Western and Asian countries for its anti-inflammatory properties [10]. According to some reports, the extract of this plant possesses some pharmacological activities which include antioxidant action, anti–inflammatory, antibacterial, antifungal, and antiviral properties [11]. Results of one clinical trial showed that *calendula officinalis* was highly effective in the prevention of acute dermatitis in patients with cancer undergoing postoperative irradiation [12]. It was observed that this plant has cytotoxic effect on tumor cell lines *in vitro* and anticancer activity *in vivo*[13]. According to the mentioned properties for *calendula officinalis* such as anti-inflammatory, anti bacterial, and antioxidant effects and considering the pathobiology of OM, it would be interesting to evaluate its effect on radiation-induced OM. Based on these concepts; this study was designed to determine the effect of calendula flowers extract mouthwash as gel formulation in the OM of head and neck cancers radiotherapy.

## Methods

### Patient selection

In this study forty patients (20 males and 20 females) with a diagnosis of head and neck cancers from Shahid-Rajaie radiotherapy center (Babolsar city, Northern Iran) were recruited. The patients with proven squamous cell cancers of head and neck were randomly divided into two equal groups either received calendula extract mouthwash or placebo (control group). Patients receiving curative radiotherapy or chemoradiotherapy were informed to take oral mouth wash calendula 5 ml b.i.d (12 hours interval). Informed consents were taken from the patients and they were instructed for oral hygiene care, avoiding spicy foods and smoking.

Patients received radiotherapy or chemoradiotherapy with a curative intent. In chemoradiotherapy groups, patients treated concurrent radiotherapy and chemotherapy,receiving cisplatin (100 mg/m2) with or without 5-fluroracil (5-FU) on the first 3 days of the first week then repeated every 3 weeks during treatment period. They were treated using cobalt-60 at 80 cm SSD. Two parallel-opposed lateral fields and an anterior lower neck field were used in daily treatment with a mid-plane radiation dose of 200 cGy to a total of 6000 –7000 cGy in 30–35 treatment sessions. The spinal cord dose was restricted to be <4600 cGy. A boost in dose of 1600–2400 cGy was given to residual disease at the primary site. Shielding blocks were used individually in cases of need. Planning target volume received at least 6000 cGy total radiation dose (200 cGy-5 days/week), with the entire of oropharynx and oral cavity at least 3 cm anterior to the retromolar trigone (no less than one third of the oral cavity) in the primary beam and Karnofsky Performance Status (KPS) >70 were the inclusion criteria.

Exclusion criteria included: history of head and neck radiotherapy, patient’s refusal, comorbid disease (diabetes mellitus, hypertension, collagen vascular disease, systemic infection, cardiac disease, etc.), poor oral hygiene [infection (viral, bacterial or fungal), active gingivitis or oral ulcer] and concurrent medications.

### Study design

This investigation was conducted as a placebo-controlled clinical trial. The protocol was approved by Ethics Committee of Babol University of Medical Sciences, Babol, Iran and it was registered in Iranian Registry of Clinical Trials (http://www.irct.ir) with ID No: IRCT201106076734N1. The informed consented patients were divided randomly into two groups of either *calendula* or placebo (20 patients in *calendula* group, and 20 patients in placebo group). To consider probable gender effect,we allocated 10 men and 10 women in both *calendula* and placebo groups. Also to ensure reliable outcomes, the patients’ tumor sites were matched by a radiation oncologist. Radiotherapy was implemented during 30 to 35 sessions in 7 weeks period with an accumulative dose of 6000 to 7000 cGy. From the beginning of radiotherapy, patients were given either placebo or 2% *calendula* extract mouthwash as a gel formulation. Drugs were administered two times daily, each time 5 ml to be held for at least one minute in oral cavity. This investigation was a double blind study. Calendula and placebo mouthwashes had a same taste, shape and odor. The OM severity in all patients was measured using Oral Mucositis Assessment Scale (OMAS) scores. It is a validated reliable semi quantitative tissue injury scoring scale, with demonstrated intra and inter-observer reproducibility [14]. This scoring system was introduced by Sonis (15) as a simple method to accurately evaluate the mucositis. Oral cavity is divided into 9 regions: hard and soft palate, floor of mouth, upper and lower lips, left and right buccal mucosa, left and right ventral and lateral tongue. All regions of oral cavity had been assessed for erythma and ulcer.

Erythema considered as: 0 point (no erythema), 1 point (mild to moderate erythema) and 2 points (severe erythema). For ulcers as follows: 0 point (no ulcer), 1 point (ulcer <1 cm), 2 points (ulcer 1–3 cm) and 3 points (ulcer >3 cm).

Oropharyngeal mucositis was evaluated by two physicians (a radiation oncologist and a dentist), using the OMAS. Indices were taken weekly during the treatment period. The maximum and minimum scores were 45 and 0, respectively [15]. Considering the treatment period of 7 weeks, 6 indices were provided for each patient.

### Mouthwash preparation

Dry and pulverized *calendula officinalis* flowers were provided from local resources and it was identified and authenticated by herbal section of Department of Pharmacology, Faculty of Medicine, Babol University of Medical Science, Babol, Iran and a voucher specimen (No: CS_00236) was deposited.

Extract of *Calendula* was made by maceration in ethanol 70% for a 72 hour period. In order to prepare the vehicle of the mouthwash we used following ingredients: Carboxymethyl cellulose (CMC) (15 g), glycerin (25 ml), methyl paraben (1.5 g), prophylparaben (0.2 g), ethanol 95^o^ (10 ml), and distilled water. The alcohol concentration in the final solution was one percent. The vehicle as the base of mouthwash was prepared as follows: first CMC was dissolved in about 250 ml distilled water, then distilled water was added gradually till the primarily volume reached 800 ml, then methyl and prophylparaben were dissolved in glycerin in a temperature of 70 to 100°C. Afterwards 100 ml of primary gel (CMC + DW) was added to them, and then, 10 ml of ethanol in 95° was added. The present solution was mixed to primary gel and remaining distilled was added to this mixture till to reach a maximum volume 1000 ml. After the preparation of mouthwash base, we added 20 g *calendula* extract for a total volume of 1000 ml, and mixed it till the mixture became uniform, in order to provide 2% *calendula* mouthwash. The placebo mouthwash is the mouthwash base which has been matched in odor, shape, and taste to *calendula* mouthwash.

### HPLC

Quercetin (main flavonoid aglycon) standard was purchased from Sigma Chemical Co. (St. Louis, MO,USA). Standard stock solution of this aglycone (20 μml^-1^) was prepared in methanol HPLC grade as a solvent and the linearity of peaks area to concentration (r = 0.983) was calculated using regression analysis. All solvents used for chromatographic purposes were of HPLC grade. HPLC was performed using a Knauer Smartline Liquid Chromatography System (Knauer, Germany) that consisted of a four-channel pump, UV/VIS detector and an injection valve, to which a 20 μl sample loop was attached. Data acquisition and processing were performed on a personal computer using Chromgate software. Sample injections were performed using a 100 μl syringe (Hamilton, Reno, Nevada, USA). The column was a Prontosil #60-5, C18 H column that was 4.6 mm in diameter and 250 mm in length. A purification system (HastaranTeb Co, FNR12, Tehran, Iran) was used for the filtration (deionization) of water. Chromatographic analysis was carried out using a single-column isocratic reverse-phase method. The mobile phase consisted of a 40:60 mixture of methanol and water containing 0.5% orthophosphoric acid. A flow rate of 0.8 mL/min was used. The analytes were detected by UV absorption at 290 nm. The standard curve of quercetin was prepared using standard solutions of different concentrations (20, 10, 4, 2 and 1 μgml^-1^). The volume for each injection was 20 μl.

*Sample Preparation for HPLC analysis:* The plant flowers (100 mg) with 6 ml 25% hydrochloric acid and 94 ml methanol was extracted and filtered through a 13 mm, 2-μm pore, using Whatman filter. The filtrate was finally diluted with methanol in the ratio 1:2 and 10 μl of this solution was injected for HPLC analysis. Each extract was diluted and injected in five repeats.

### Determination of total antioxidant activity

Total antioxidant activity was estimated by a FRAP (ferric reducing antioxidant power) assay [16]. Five different concentrations of the calendula extracts (0.1, 0.2, 0.4, 0.5, 1%) were used for this test. Principle of this method is based on the reduction of a ferric-tripyridyltriazine complex to its ferrous, colored form in the presence of antioxidants and the absorbance was read at 593 nm with a single beam Jenway UV/Visible spectrophotometer (UK) and compared to a blank. The final result was expressed as the concentration of antioxidant with a ferric reducing ability equivalent to that of 1 mmol/l FeSO4 [17].

### Total phenols determination

Total phenolic contents were evaluated using Folin-Ciocalteu reagent [18]. A dilute solution of the extract (0.5 ml of 1:100 mg ml-1 methanolic extract) or gallic acid (phenolic standard compound) was mixed with the reagent (5 ml, 1:10 diluted with distilled water) and aqueous Na2CO3 (4 ml, 1 M). After 15 min standby period, the total phenol contents were assayed using colorimetry at 765 nm. The standard curve was obtained using 0, 25, 50, 100, 150, 250 and 500 mg l-1 solutions of gallic acid in methanol:water (50:50, v/v). Total phenolic contents were shown as mg/g (of dry mass) of gallic acid equivalent, which is a common reference compound.

### Total flavonoids determination

Total flavonoid contents were determined using aluminum chloride colorimetric method [19]. Calendula methanolic extract (0.5 ml of %1 extract) was mixed with 1.5 ml of methanol, 0.1 ml of 10% aluminum chloride, 0.1 ml of 1 M potassium acetate and 2.8 ml of distilled water. Then the mixture was left at room temperature for 30 min; the absorbance of the reaction mixture was measured at 415 nm. Different quercetin solutions at concentrations 6.25, 12.5, 25, 50 and 100 mg l-1 in methanol were prepared to obtaining standard curve.

### Statistical analysis

Data were analyzed by using *t*-test, Mann–Whitney *U* test, one-way ANOVA and repeated measures ANOVA tests. The *p* values of less than 0.05 were considered as the statistical significant difference.

## Results

**Figure 1:**
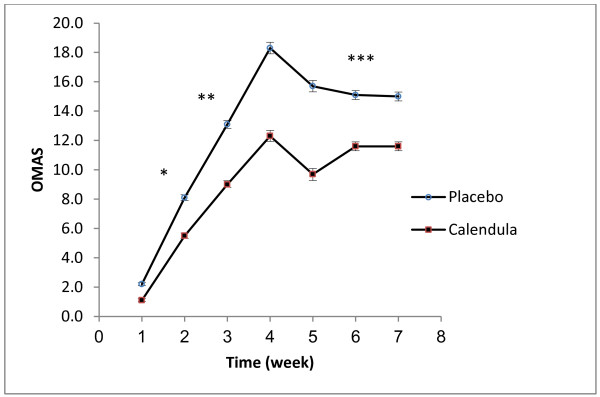
**Mean (±SEM) of OMAS scores changes by time (week) in placebo and calendula treatment group.** * p = 0.019, ** p < 0.0001, *** p = 0.031.

**Figure 2:**
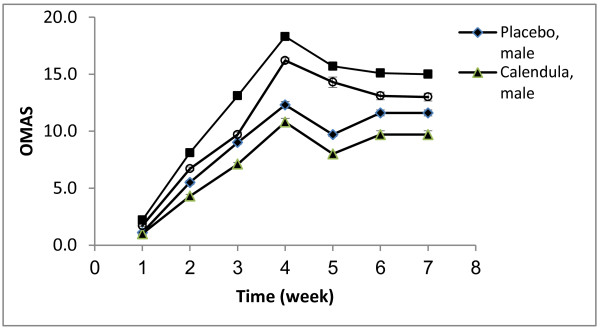
Mean (±SEM) of OMAS scores changes by sex and time (week) in placebo and calendula treatment group.

**Figure 3:**
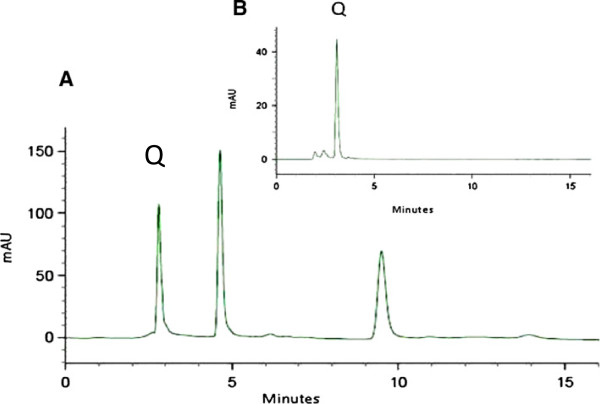
**HPLC fingerprint obtained from hydroalcolic extract of *calendula officinalis *flowers (A), compared to quercetin standard [Q, 20 microgram/ml] (B).** Method characteristics: methanol (40%), 0.5% aqueous solution of orthophosphpric acid (0.6%), flow rate, 0.8 ml/min, detection at 290 nm.

In the pcacebo group , six patients were excluded : Four patients had left the study , two patients had severe mucositis and treated with another drugs. In the calendula group ,three patients were exclude that had left the study. Statistical analysis was performed on 20 and18 patients in calendula and placebo groups respectively (Additional file 1). Thirty eight patients with mean age 54.1 (range 46–72) completed the study. Individual characteristics of the patients are detailed in Table 1. None of the patients in *calendula* group underwent medication for OM severity and radiotherapy was not ceased for this reason. In *calendula* group three patients did not show OM in the whole treatment period. OM intensity was measured according to OMAS scores at the end of each week for both *calendula* and placebo groups. OMAS scores were significantly lower in *calendula* group compared to placebo at week 2, 3 and 6 of the study (Figure 1). According to repeated measures ANOVA test, the differences between OMAS of *calendula* and placebo during the weeks of evaluation were statistical significant (p < 0.001) and OM intensity was significantly decreased in *calendula* compared to placebo group (*p* = 0.048). Although OM intensity was higher in women, but in both group of genders *calendula* had better results compared to placebo (Figure 2). Quercetin concentration as a main flavonoid content was measured. According to the standard curve, the mean ± SD of quercetin concentration in the extract was 19.41 ± 4.34 mg/l (%*RSD = 15.33*). HPLC fingerprint of hydroalcolic extract of *calendula officinalis* is shown in Figure 3. The flavonoid contents of *calendula* flowers extract based on quercetin equivalent concentration (the standard curve equation: y =0.007× + 0.3337, r = 0.998) was 76.66 ± 23.24 mg/g.

**Table 1:** Comparison of the patients’ individual results in among two treatment groups

**Treatment groups**	**Placebo**	**Calendula extract**
**Parameters**		
Number of patients	20	20
*Male*	*10*	*10*
*female*	*10*	*10*
Mean age	52.8	52.2
Tumor location		
*Nasopharynx*	*10*	*11*
*Oral Cavity*	*7*	*6*
*Salivary glands*	*1*	*-*
*Tonsil*	*2*	*3*
Radiotherapy alone	4	3
Chemoradiotherapy	16	17
Total	20	20

The FRAP results for ferric reducing ability in five concentrations (0.1, 0.2, 0.4, 0.5, and 1%) of *calendula* extracts were 565.5 ± 126.5, 1059.2 ± 305.1, 1714.3 ± 318.5, 1877.3 ± 381.8 and 2353.4 ± 56.5 μM, respectively. Total phenol using gallic acid concentration (the standard curve equation: y =0.005× + 0.001, r = 0.999) was 313.40 ± 6.52 mg/g of gallic acid equivalent in the extract of *calendula officinalis*.

## Discussion

When biological changes induced by radiotherapy is being initiated, an extensive damage is seen to result in destruction of intact mucosa [20]. In the present study three patients of *calendula* group didn’t demonstrate OM after radiotherapy in the whole treatment period.

Most interventions have been proven to be ineffective and current clinical management of OM is mainly palliative and focused on pain control [21]. Up to date, no study has investigated the effect of *calendula officinalis* on radiation-induced OM. Many beneficial activities reported for the plant are mainly related to the contents of various secondary metabolites such as polyphenols, cartenoids, triterpenes and essential oils [22]. Several *calendula* therapeutic properties such as, anti-inflammatory, anti-tumorogenic [23,24] have been reported. In addition, *in vitro* anti-microbial activities of its oils have been proven [25]. Regardless of bacterial counts which may increase over 300% compared to baseline, increasing gram negative bacteria that is seen during ulceration needs to be re-established by normal bacterial proportions for spontaneous ulcer resolution [26]. Antioxidant activity and wound healing properties of *calendula* has been reported [27]. According to the present results, antioxidant activity of the extract was confirmed. It has been demonstrated that plant polyphenols such as flavonoids are a most important active natural products possessing antioxidant activity. This effect is considerable on human health. The flavonoids which contain hydroxyl groups are responsible for the radical scavenging [28-30] or chelating [31-33] effects. These agents accompanying with several endogenous antioxidants may play an prominent role in maximum protection against oxidative stress induced by high level of reactive oxygen species (ROS) in the body. On the other hand, phenolic compounds are a class of antioxidant agents which act as free radical terminators [34,35]. The ROS play a causative role in several degenerative diseases, for example heart disease, atherosclerosis, hepatotoxicity, inflammation, tumor promotion, and cancer [36-38]. In this study quercetin as a flavonoid with anti-inflammatory effect [39-41] has been assayed in the *calendula* flower extract and antioxidant capacity was measured using FRAP test as well.

According to our investigation, the high contents of the flovonoid and phenolic phytochemicals in *calendula officinalis* and its antioxidant activity can support its high radical scavenging activity and following protective effect in radiotherapy- induced OM. Initiation phase of radiotherapy or chemotherapy-induced injury is a critical first step in the development of mucositis in which clonogenic cell death and the production of ROS by injured cells are the most dominant components. The initiation phase is a rate-limiting step and by delaying or stopping it, it can be prevented or minimized by regimen-related injury [42]. Considering the antioxidant properties of *calendula*, it may act against ROS and prevents or delays the initiation phase. In one study which evaluated laser activated *calendula* extract (LACE), the LACE showed to be capable *in vitro* inhibition of tumor cell proliferation, from 70 to 100% when tested on a wide variety of human and murine tumor cell lines. According to this study, LACE has immune-modulatory activities [5,23,43]. There is very few safety concerns related to *calendula*. However, systemic administration of *calendula* products should be avoided during early stages of pregnancy due to its ability to stimulate menstruation, also allergic hypersensitivity may cause a problem for individuals sensitive to other members of the plant family *asteraceae*[44]. It has been shown that *calendula* extract toothpaste could effectively reduce plaque and bleeding indexes in moderate to severe gingivitis. It has been suggested that anti-inflammatory, antibacterial and antioxidant properties of *calendula* may be responsible for mentioned effects on gingivitis [45]. On the other hand, it has been reported *calendula* flowers extract as an ointment is effective in relieving pain of cracked or tender nipples [46]. Duran *et. al*. (1996), showed positive preliminary results for the administration of *calendula* ointment in the treatment of venous leg ulcers [47]. It has been demonstrated that *calendula* as a cream preparation protected skin from irritant contact dermatitis caused by exposure to sodium lauryl sulfate [48]. And as earlier mentioned, *calendula* was highly effective in the prevention of acute dermatitis in patients with cancer undergoing postoperative irradiation [12]. Although, dermatitis and OM have different pathobiology profiles, among reviewed clinical trials the mentioned study was the closest one to our investigation. Generally there are few studies concerning effects of herbal extracts on OM. The fact that *calendula* mouthwash could not completely prevent OM can be explained by the complicated pathobiology of OM. It is also suggested using *calendula* preparations in high dose of in order to be more effective, and it perhaps need to be applied for a longer duration of time, even though different protocols for herbal extraction should be considered as a factor when evaluating its effects. In the present study, alcohol containing mouthwash was used. Although the concentration of alcohol in final solution was lower than to induce injury to the oral mucosa, the irritant effect of alcohol may be questionable. In other words, it has been found that chlorhexidine mouthwash containing alcohol, can irritate the oral mucosa and it is effective in reducing the duration of pain associated with oral mucositis induced by chemotherapy (49). Regarding to comparison of OM in two genders, it has been demonstrated that its intensity is higher in females than males. Severe stomatitises and radiotherapy induced OM, has been reported in women [49]. This may be due to physiological differences between two sexes but its exact mechanism remains to be elucidated.

Finally, it is concluded that *calendula officinalis* is effective on decreasing OM intensity but cannot completely prevent its occurance. All patients could tolerate it without any significant side effects (i.e., nausea and vomiting). Furhter investigations are recommended to elucidate the optimal dose and administration frequency to have better outcomes in radiotherapy-induced OM.

## Competing interest

The authors declare that they have no competing interests.

## Authors’ contributions

NB: physical exam and study design; DM: physical exam and study design and manuscript writing; MK, MB: data collection; FV: English writing; YM: English writing and grammar correction; AB: statistical analysis; MTK, AK: lab tests examiner; MP: biochemical analysis; AAM: study design and manuscript writing. All authors read and approved the final manuscript.

## Supplementary Material

Additional file 1 CONSORT 2010 flow diagram. Click here for file
