# Supplementary material for: Antioxidant capacity of calendula officinalis flowers extract and prevention of radiation induced oropharyngeal mucositis in patients with head and neck cancers: a randomized controlled clinical study
Source: Daru. 2013 Mar 7;21(1):18. doi: 10.1186/2008-2231-21-18 (PMC3623793; doi:10.1186/2008-2231-21-18)
Supplement: Additional file 1 — CONSORT 2010 flow diagram. [file 2008-2231-21-18-S1.doc]

**CONSORT 2010 Flow Diagram**

**Allocation**

**Analysis**

**Follow-Up**

**Enrollment**

Assessed for eligibility (n= 48 )

Excluded (n=8 )

♦  Not meeting inclusion criteria (n=4 )

♦  Declined to participate (n= 3 )

♦  Other reasons (n= 1 )

Analysed (n=20 )
♦ Excluded from analysis (give reasons) (n=0 )

Lost to follow-up (give reasons) (n= 0 )

Discontinued intervention (give reasons) (n= 0 )

Allocated to calandula (n= 23 )

♦ Received allocated intervention (n=20 )

♦ Did not receive allocated intervention (Lack of patient interest) (n=3 )

Lost to follow-up (discontinuation of treatment) (n=0 )

Discontinued intervention (severe mucositis-treatment with another drugs) (n= 2 )

Allocated to placebo (n=26 )

♦ Received allocated intervention (n=22 )

♦ Did not receive allocated intervention (Lack of patient interest) (n= 4 )

Analysed (n= 20 )
♦ Excluded from analysis (treatment interruption) (n=0 )

Randomized (n= 40 )
